# Effect of Four Weeks of Home-Based Balance Training on the Performance in Individuals with Functional Ankle Instability: A Remote Online Study

**DOI:** 10.3390/healthcare9111428

**Published:** 2021-10-23

**Authors:** Mohammadreza Seyedi, Hadi Nobari, Hamed Abbasi, Davood Khezri, Rafael Oliveira, Jorge Pérez-Gómez, Georgian Badicu, José Afonso

**Affiliations:** 1 Department of Sport Injuries and Corrective Exercises, Sport Sciences Research Institute, Tehran 1587958711, Iran; Seyedi@ssrc.ac.ir; 2 Department of Physical Education and Sports, University of Granada, 18010 Granada, Spain; 3 HEME Research Group, Faculty of Sport Sciences, University of Extremadura, 10003 Cáceres, Spain; jorgepg100@gmail.com; 4 Department of Sport Biomechanics and Technology, Sport Sciences Research Institute, Tehran 1587958711, Iran; daividkhezry@gmail.com; 5 ESDRM-IPS—Sports Science School of Rio Maior–Polytechnic Institute of Santarém, 2040-413 Rio Maior, Portugal; rafaeloliveira@esdrm.ipsantarem.pt; 6 Research Centre in Sport Sciences, Health Sciences and Human Development, Quinta de Prados, Edifício Ciências de Desporto, 5001-801 Vila Real, Portugal; 7 Life Quality Research Centre, Complexo Andaluz, Apartado 279, 2001-904 Santarém, Portugal; 8 Department of Physical Education and Special Motricity, Transilvania University of Brasov, 500036 Brasov, Romania; georgian.badicu@unitbv.ro; 9 Centre for Research, Education, Innovation and Intervention in Sport, Faculty of Sport, University of Porto, 4099-002 Porto, Portugal; jneves@fade.up.pt

**Keywords:** injuries, home-based exercises, functional skills, prevention, sport

## Abstract

The purpose of the current study is to evaluate the effect of 4 weeks of home-based balance training (HBBT) on the performance of individuals with functional ankle instability (FAI) in daily activities and sports. Thirty college students diagnosed with FAI and with a mean weight of 79.8 ± 3.4 kg, height of 182.5 ± 5.1 cm, age of 23.5 ± 1.2 years, and instability score of 20 ± 2.3 were selected to participate in this study and were randomly divided by computer-generated methods into two groups: the HBBT group and the control group (CG), each consisting of 15 subjects. The HBBT group performed the program at home for 4 weeks, while the CG was non-exercise. Before and after the 4 weeks of exercise program, a form containing the foot and ankle ability measure for daily activities and sports was completed by the individuals. For data analysis, intra- and inter-group comparisons were performed using paired and independent sample *t*-tests, respectively, at a significance level of *p* ≤ 0.05. The results showed that 4 weeks of progressive HBBT were sufficient to significantly improve the measurement of the ability of ankle and foot function in individuals with FAI, even with a total volume of only 60 min per week. Accordingly, it is suggested that individuals with FAI can benefit from short-term HBBT programs, which are simple yet powerful enough to promote improvements in daily activities.

## 1. Introduction

The ankle joint is one of the most commonly affected joints during sports and daily life activities [1]. The clinical importance of ankle sprain emerges from its impact on daily activities and its recurrence, which is associated with joint diseases and trauma, with up to 74% of people developing chronic ankle instability [2]. Research has shown that degenerative changes and rheumatoid arthritis are common complications associated with the recurrence of ankle sprain [1,3,4]. Ankle injuries also affect sport performance. Indeed, they have been shown to cause athletes to be away from sports fields, decreasing their ankle range of motion and impairing daily activities such as walking and running [5]. It should be noted that ankle sprains are involved in about 20% of all sports injuries [3]. In athletes, injuries to the lateral ligaments of the ankle joint are the most common, representing about 85% of the total sprains [6].

In more than 70% of people who suffered from lateral ankle sprain, the residual effects remained for up to 18 months after the initial injury [7,8]. Recent studies found a significant association between ankle injury, recurrence and subjective feeling of giving away [9,10]. Early signs of this damage include pain, muscle weakness, impaired proprioception and recurrent ankle sprain [11]. Ignoring foot and ankle injuries or inadequate rehabilitation can result in the recurrence of problems such as mechanical and functional instability [12]. Freeman [11] first defined ankle instability as the tendency of the foot to incur recurrent ankle sprains. Functional ankle instability (FAI) is a common complication in 15 to 60% of cases following the initial sprain [4,13,14]. Special clinical tests, such as the anterior draw test, can be used to assess mechanical ankle instability. FAI is difficult to measure and primarily diagnosed through patient-reported outcome measures [15]. Moreover, several researchers have used the foot and ankle ability measures (FAAM) questionnaire to assess lower extremity function in patients with FAI [16]. For example, Clark and Borden [17] used the same questionnaire and reported that the levels of perceived instability of the ankle joint significantly increased after 4 weeks on the balance board. Mckeon et al. [18] also showed that the 4 week dynamic balance exercises can lead to improvements in the self-reported performance of non-athletes with FAI suffered during daily activities and can be measured using the Questionnaire Disability Index of the ankle and foot. This questionnaire has shown to be valid and has high intersession reliability [19].

The home-based exercise programs have been reported to have their problems, such as reduced adherence rates and low compliance with the planned activities. However, among the available rehabilitation programs, the use of home-based exercise programs for the rehabilitation of affected individuals is on the rise and can be particularly important in the context of a worldwide pandemic such as COVID-19 [20]. The use of home-based exercise programs can empower patients to become more active in their own recovery [21] while enabling a time- and cost-effective practice [22]. Home-based programs should be simple to implement, using easy-to-understand exercises that require minimum material and costs while at the same time being safe for the patients. In this vein, studies have been conducted on the effect of home-based exercise on proprioception and postural control [23]. The study of changes in the functional limitation of people with ankle instability is important, but, to our knowledge, only one study to date has assessed the feasibility of home-based programs to ameliorate FAI [23]. The aim of this study, therefore, was to investigate the effect of 4 weeks of home-based balance training (HBBT) on the performance of people with FAI in daily activities and sports.

## 2. Materials and Methods

### 2.1. Participants 

The study population consisted of young male university students with FAI who were playing in volleyball, basketball and handball teams. All participants in the study were male students who had regular training over the last three years. They were also members of collegiate teams. In this study, 30 subjects were purposefully selected with a mean and standard deviation (SD): body mass of 79.8 ± 3.4 kg, height of 182.5 ± 5.1 cm, age of 23.5 ± 1.2 years and instability score of 20 ± 2.3. Thereafter, they were randomly divided into two groups by computer-generated methods: (1) the HBBT group and (2) the control group (CG), each with 15 subjects. They did not differ in baseline values between the two groups (Table 1), so groups were homogeneous [24].

Subjects had to match the International Ankle Consortium’s standard inclusion and exclusion criteria [25].

#### 2.1.1. Inclusion Criteria

I. At least one severe ankle sprain in the past;
II. The first sprain must have happened at least 12 months before enrolling in the trial;
III. The initial sprain has to be accompanied by inflammatory signs (i.e., pain, edema and so on); at least 1 day of targeted physical activity must have been disrupted by the first injury;
IV. The most recent injury must have occurred at least 3 months before enrolling in the study;
V. Previous ankle joint injury with giving way, recurrent sprain or feelings of instability;
VI. In the 6 months leading up to study enrolment, participants must report at least two occurrences of giving way;
VII. Experiencing a recurring sprain, which is defined as two or more sprains on the same ankle;
VIII. Instability in the ankle joint;
IX. Cumberland Ankle Instability Tool 41: <24 confirmed self-reported ankle instability.

#### 2.1.2. Exclusion Criteria

I. A history of past musculoskeletal surgery in either lower extremity limb;
II. A history of a fracture in one of the lower extremity limbs that necessitated a realignment;
III. Acute damage to the musculoskeletal structures of other joints in the lower extremity in the past 3 months that resulted in at least 1 day of missed physical activity;
IV. Ankle anterior drawer test was positive;
V. Only one training session was missed.

**Figure 1:**
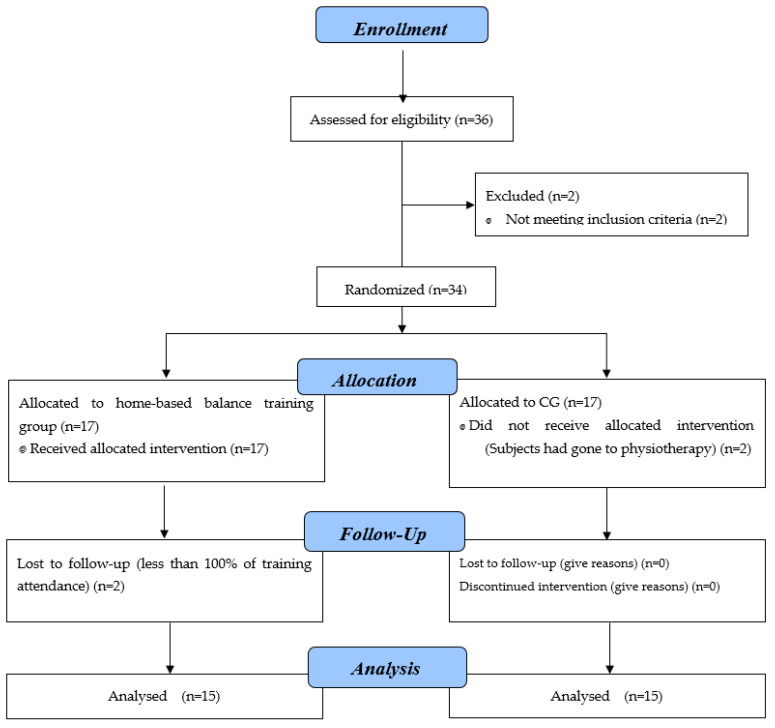
CONSORT flow chart of participants for recruitment, application, follow-up and analysis.

The selection of subjects with FAI was conducted through a clinical anterior drawer test of ankle, and scores were obtained using the FAI questionnaire (i.e., Cumberland Ankle Instability Tool) [13,16]. The questionnaire, which consists of nine multiple-choice questions, provides information on the extent and onset of pain and symptoms associated with sprained ankle injuries as well as the biomechanical and motor mechanisms that lead to symptoms. A lower score indicates greater severity of the injury as experienced by the person answering. The validity and reliability of this questionnaire in assessing the severity of ankle sprain injury have been confirmed by previous studies [26]. The functional stability score of the ankle is between 0 and 30; the score range of 27 to 30 represents good health of the ankle, while a score representing ankle instability ranges from 0 to 27. In scores between 0 and 27, lower scores in subjects indicates more severe ankle instability [27]. In addition, none of the subjects had any history of vision, hearing or neurological defects. Subjects signed a consent form to participate in this study. The Ethics Committee of the Sport Sciences Research Institute (IR.SSRI.REC.1399.905) gave approval before the start of the study, and during the study we followed the recommendations on human research based on the Helsinki Declaration. The CONSORT diagram of participant recruitment, allocation, follow-up and analysis is indicated in Figure 1.

### 2.2. Sample Power

The *t*-test family sample power was calculated a priori to compute achieved power: α error prob level = 0.05; effect size = 0.5 [18]; 1 − β error prob = 0.84 by the G*Power tool. There was an 84.8% actual power with the present analysis of 30 subjects [28]. We carried out this analysis with G*Power software (University of Düsseldorf, Düsseldorf, Germany).

### 2.3. Tools and Measurements

All anthropometric assessments (i.e., height and body mass) were based on recommendations in previous studies [29,30] and questionnaires in order to obtain background information about age and physical activity completed. Participants also completed a form containing an evaluation of the ability of the foot and ankle in daily activities and sports (i.e., FAAM) in order to assess the self-reported performance before and after the study. The form used to evaluate the foot and ankle ability was used to measure musculoskeletal disorders of the lower extremities, ankles and feet [16]. This questionnaire was designed to assess the physical function self-report in patients with ankle instability [16]. The scale used to evaluate foot and ankle ability in daily activities included 21 cases related to activities of daily living, while the scale for the evaluation of foot and ankle ability in sporting activities included 8 cases. These scales were used to assess injuries that resulted from an ankle disability which was associated with physical activity and sports. The reliability of the questionnaire was reported for daily physical activity and sport as 0.89 and 0.87 [16]. Values for the Persian version of this questionnaire were reported for daily physical activity and sport as 0.97 and 0.94 [31]. The scale used for evaluating foot and ankle ability in daily activities had an overall score of 84%; however, the questionnaire score was related to the overall score of 32 for physical activities. At the end of the questionnaire, each individual obtained a score between 0 and 100% of his/her current level of performance in daily activities and sports [31].

Participants in the exercise group performed a progressive HBBT exercise program for 4 weeks in 3 weekly sessions, with ~20–30 min duration per session. Both groups kept their regular sports practice during the course. Table 2 and Table 3 show the progress of the exercises and the program. The exercises were performed only on the injured leg and were used to improve the exercises of changes in hand placement, vision system and surface type [32]. These exercises were designed based on the reports of previous studies [23,33,34]. 

**Figure 2:**
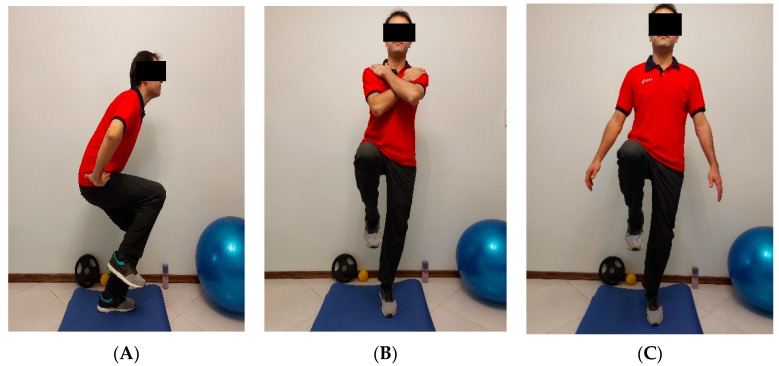
How to perform the move of the single leg squat airex, eyes closed, hands on the hips (**A**); single leg stance, airex, eyes closed, arms crossed (**B**) and single leg stance, airex, eyes closed, arms free (**C**).

Before the start of the exercises, the method of performing the exercises was explained to the participants. In a one-on-one training session, a sample of the home exercise group was taught in person by a sports injury specialist, and educational videos were prepared in each of which a 20 min session was taught to individuals. The HBBT group was handed over to perform the exercises accordingly. Each subject received a foam for exercises. The foam was semi-rigid and composed of polyurethane, with a size of 0.2 × 5.1 × 2 m. In addition, during all training sessions subjects were contacted via video call to be motivated and ensure that the exercise was performed correctly. An example of the exercises can be seen in Figure 2.

### 2.4. Statistical Analysis 

Data were obtained and analyzed using SPSS software version 20 (IBM, Armonk, NY, USA) and a lower alpha level of 0.05. Descriptive statistics are reported as mean ± SD. The Shapiro–Wilk test and Levene’s test were used to check the normality and homogeneity of variables of data, respectively. Thirty of the initial set of 34 enrolled participants (i.e., 88.2% of the sample) completed the trial. Meanwhile, dropouts were the same in both groups (*n* = 2 in each) and for reasons unlikely to be related to the interventions. Therefore, we considered that a per protocol analysis would be suitable and would not deviate meaningfully from an intention-to-treat analysis. A factorial ANOVA with repeated measures was performed on assessment questionnaire parameters. The intra-group factor took into account time (pre- or post-test), while the inter-group factor took into account groups (HBBT or CG). Each group was treated with a one-way repeated-measures ANOVA with a Bonferroni post hoc analysis when a significant time–group interaction was observed. For calculation to define the magnitude of pairwise comparisons for pre- and post-test, we used an effect size of Hedge’s g type with a 95% confidence interval and defined as trivial (<0.2), small (≥0.2), moderate (≥0.5) or large (≥0.8) [35].

## 3. Results

There were significant main effects of time and group-by-time interactions for change times in the FAAM for daily activities (*p* ≤ 0.001, *F* = 20.3, *η_p_*^2^ = 0.42 and *p* = 0.001, *F*= 14.9, *η_p_*^2^ = 0.35, respectively). Post hoc analysis revealed that the FAAM for daily activities was significantly (*p* ≤ 0.001, *g* = 2.1) greater after intervention compared to before in HBBT. The CG showed a non-significant (*p* = 0.65, *g* = 0.5) trend for post-test compared to pre-test.

In addition, significant changes in the FAAM for daily sports were found for the main effect of time (*p* ≤ 0.001, *F*= 107.8, *η_p_*^2^ = 0.80) and group-by-time interactions (*p* ≤ 0.001, *F* = 79.9, *η_p_*^2^ = 0.74). Post hoc analysis revealed that the FAAM for daily sports was significantly (*p* ≤ 0.001, *g* = 4.5) greater at post-test compared to pre-test in HBBT. However, it was not significant (*p* = 0.32, *g* = 0.2) for the CG. Moreover, the results of pairwise comparisons after intervention showed that the HBBT group outstripped the CG in the FAAM for daily activities and sports (*p* ≤ 0.001). Consequently, in practice, the proposed training plan efficiently assisted individuals who participated in this study to regain their function in daily activities and sports to some extent (Table 4).

## 4. Discussion 

The results of this study showed that as few as 4 weeks of a progressive HBBT program significantly improved the performance of daily activities and sports, which was measured using a questionnaire [31] that was designed to evaluate the ability of the ankle and foot in athletes with FAI. The performance of the CG after the test did not show any significant change as compared with the pre-test, using scores from two questionnaires which demonstrated that (1) the natural biological recovery was insufficient to ameliorate the situation and (2) the exercise program was effective in restoring the performance of daily activities and sports. Similar to the results of this study, Clarke and Borden [17] showed that the implementation of balance exercises on a wobble board for 4 weeks caused a significant increase in the levels of performance in the questionnaire designed to evaluate ankle and foot ability [17]. Mckeon et al. [18] also showed that the performance of 4 weeks of exercises improved the performance of dynamic balance, measured by the disability index of the ankle and foot in daily activities and sports in people and athletes with FAI [36,37,38].

Research seems to consistently show that a short-term program is effective in ameliorating the symptoms, in comparison with controls not engaged in a specific rehabilitation program. This shows that such programs are effective in accelerating the natural, biological course of events. The possible cause of improvement in performance levels of the participants in balance exercises can be attributed to increased sensitivity of the sensorimotor systems as a result of the adoption of this practice [18]. Similarly, changes in the motion programs of patients with FAI was one of the problems that caused delay in changing the ankle stabilizer muscles and, as such, resulted in a feedback system that caused the recurrence of sprain [8,18]. The implementation of balance exercises led to deep proprioception receptor feedback to the central systems to correct the action programs, which consequently led to activities that were accurate and timely (feed-forward) and would prevent recurrent ankle sprain. As a result, one of the possible reasons for the increase in their performance may be the refinement of their motor programs [39].

The results also showed that 4 weeks of progressive HBBT, measured using a questionnaire, significantly improved ankle and foot function in daily activity (*g* = 2.1), with a value difference of 12.3, and sport (*g* = 4.5), with a value difference of 25.3 with FAI. In addition, HBBT also improved the ability of the sensorimotor system to overcome the limitations caused by increased instability. Given the importance of the chronic effects of exercise, it is suggested that future studies be conducted on the lasting effect of balance training on various factors, such as balance and posture, e.g., perhaps including a no-intervention period followed by a retention test.

As shown by the results, using HBBT successfully contributes to the treatment of ankle instability. This leads to an increase in the cost- and time-efficiency of the treatment process and helps to reach high demands of treatment as well. Therefore, for better rehabilitation, it is recommended to use these exercises under supervision.

### Limitations

The use of self-reported questionnaires may be a limitation, and the prescribed program may have promoted a placebo effect that possibly influenced the self-assessments. More objective testing of function, made by experienced clinicians blinded to the intervention, would provide a more robust assessment. It is also possible that the merits relied more on the extra exercise in general (i.e., on the extra 3 weekly sessions of 20–30 min of exercise for 4 weeks) and not on the specific prescription of our exercise program. Future studies should attempt to equate training volume between the groups to better scrutinize this feature.

## 5. Conclusions

These results demonstrate that even very simple and unsupervised interventions can go a long way and be very useful for accelerating recovery from injury. Coaches in underprivileged settings can still help their athletes recover faster and better using simple, home-based exercises that are easy to teach, safe to implement and very low-cost.

## Figures and Tables

**Table 1:** Demographic characteristics of subjects in each group.

Group	*n*	Age (Years)	*p*	Weight (kg)	*p*	Height (cm)	*p*
HBBT	15	23.7 ± 1.4	0.704	81.1 ± 3.7	0.238	183.2 ± 5.4	0.357
CG	15	23.3 ± 1.2		78.6 ± 2.6		181.7 ± 4.9	

Abbreviations: CG: control group; HBBT: home-based balance training; kg: kilograms; cm: centimeters.

**Table 2:** Progressive training program for intervention group.

Exercise	Se 1	Se 2	Se 3	Se 4	Se 5–6	Se 7–8	Se 9–10	Se 11–12
A	1 (3 × 10 s)	2 (3 × 15 s)	3 (3 × 15 s)	3 + 4 (2 × 15 s)	3 + 4 (2 × 20 s)	4 + 5 (2 × 15 s)	5 + 6 (2 × 20 s)	v3 + 6 (2 × 20 s)
B	1 (3 × 10 s)	1 (3 × 15 s)	2 (3 × 15 s)	2 (3 × 20 s)	3 (3 × 20 s)	3 (3 × 25 s)	4 (3 × 20 s)	4 (3 × 25 s)
C	1 (3 × 5 rp)	1 (3 × 7 rp)	2 (3 × 5 rp)	2 (3 × 7 rp)	3 (3 × 6 rp)	3 (3 × 8 rp)	4 (3 × 5 rp)	4 (3 × 7 rp)
D	1(3 × 15 rp)	1 (3 × 15 rp)	1 (3 × 20 rp)	1 (3 × 20 rp)	2 (3 × 10rp)	2(3 × 12 rp)	2 (3 × 15 rp)	2 (3 × 15 rp)
E		1 (3 × 10 rp)	2 (3 × 10 rp)	2 (3 × 12 rp)	3 (3 × 10 rp)	3 (3 × 12 rp)	4 (3 × 10 rp)	4 (3 × 12 rp)

Numbers represent each type of exercise and duration/repeats needed in each session; Se: session, s: seconds, rp: repeats.

**Table 3:** Home-based balance training program.

Movements Training	
**A. Single leg stance:** 1. Firm surface, arms crossed with EO; 2. Firm surface, arms free with EC; 3. Firm surface, arms crossed with EC; 4. Airex, arms crossed with EO; 5. Airex, arms free with EC; 6. Airex, arms crossed with EC.	**C. Single leg squat:** 1. Firm surface, hands on the hips with EO; 2. Firm surface, hands on the hips with EC; 3. Airex, hands on the hips with EO; 4. Airex, hands on the hips with EC.
	**D. Heel raise:**
	1. Firm surface, bilateral with EO;
	2. Firm surface, unilateral with EO.
**B. Crossed leg sway:** 1. Firm surface, hands on the hips with EO; 2. Airex, hands on the hips with EO; 3. Airex, arms free with EC; 4. Airex, hands on the hips with EC.	**E. Lunge/jump exercise:**
	1. Firm surface, 45 cm to the front with EO; 2. Firm surface, 45 cm to the front and sidelong with EO; 3. Firm surface, 70 cm to the front and sidelong with EO; 4. Airex, 45 cm to the front and sidelong with EO.

EC: eyes closed; EO: eyes opened.

**Table 4:** Between- and within-group differences in FAAM scores before and after intervention.

Questionnaires	Groups	Pre-Training	Post-Training	CI95% for Difference	Hedge’s *g* (95% CI)
				Values [Lower–Upper]	Values [Lower–Upper]
**FAAM-daily**	**HBBT**	6.8 ± 79.7	3.5 *^#^ ± 91.9	12.3 [8.3 to 16.3]	2.1 [1.2 to 3.0]
	**CG**	7.7 ± 78.1	4.9 ± 81.8	3.8 [−1.1 to 8.6]	0.5 [−0.2 to 1.3]
**FAAM-sport**	**HBBT**	5.9 ± 62.9	4.5 *^#^ ± 88.2	25.3 [21.4 to 29.2]	4.5 [3.1 to 5.8]
	**CG**	4.2 ± 61.0	5.4 ± 61.9	1.0 [−2.7 to 4.6]	0.2 [−0.5 to 0.9]

Data are presented in mean ± standard deviation. FAAM-daily: foot and ankle ability measure in daily activities; FAAM-sport: foot and ankle ability measure in sports; CG: control group; HBBT: home-based balance training; Hedge’s g (95% CI): Hedge’s g effect size magnitude with 95% confidence interval. * Demonstrated significant results compared to the pre-test. ^#^ Demonstrated significant results compared to the CG.

## Data Availability

The datasets used and/or analyzed during the current study are available from the corresponding author on reasonable request.
